# The clinical effectiveness and safety of traditional Chinese medicine Jinfeng pill in adjuvant treatment of infertility with polycystic ovary syndrome: A protocol for systematic review and meta-analysis

**DOI:** 10.1097/MD.0000000000028676

**Published:** 2022-01-28

**Authors:** Ping Zhu, Jing-Zhi Guan, Qing-Chun Hai, Jing Jin, Lei Shi, Lian Hua

**Affiliations:** a Department of Obstetrics and Gynecology, Inner Mongolia Baogang Hospital (The Third Affiliated Hospital of Inner Mongolia Medical University), NO.20 Shaoxian Road, Kundulun District, Baotou, Inner Mongolia Autonomous Region, China.; b Department of Pharmacy, Inner Mongolia International Mongolian Hospital, NO.83 Daxue East Road, Hohhot, Inner Mongolia Autonomous Region, China.; c Department of Acupuncture and Massage, Inner Mongolia International Mongolian Hospital, NO.83 Daxue East Road, Hohhot, Inner Mongolia Autonomous Region, China.; d Department of Obstetrics and Gynecology, Baogang Third Hospital of Hongci Medical Group, NO.15 Qingnian Road, Kundulun District, Baotou, Inner Mongolia Autonomous Region, China.; e Department of Pharmacy, The First Hospital of Hohhot, Hohhot, Inner Mongolia Autonomous Region, China.; f College of Mongolian Medicine, Inner Mongolia Medical University, Jinshan Development Zone, Hohhot, Inner Mongolia Autonomous Region, China.

**Keywords:** adjuvant treatment, effectiveness and safety, infertility with polycystic ovary syndrome, meta-analysis, randomized controlled trial, traditional Chinese medicine Jinfeng pill

## Abstract

**Background:**

Polycystic ovary syndrome (PCOS) is the main cause of infertility in women, the essence of which is an endocrine disorder syndrome with abnormal sugar metabolism and reproductive dysfunction, and the incidence rate of about 6% of women. Traditional Chinese medicine (TCM) Jinfeng pill has achieved very good clinical results in the treatment of infertility with PCOS, but there is currently a lack of strong evidence-based medical evidence. This study uses meta-analysis method to analyze the clinical effectiveness and safety of TCM Jinfeng pill in the treatment of infertility with PCOS, hoping to provide help for the clinical treatment of infertility with PCOS.

**Methods:**

Using the computer to retrieve SinoMed, CNKI, VIP, WANFANG Database, as well as Public, The Cochrane library, Medline (Ovid SP), Embase and other foreign language databases, while manually retrieving the relevant magazine supplements, special issues, professional materials, network information, and so on. The retrieval time is from the beginning of each database to June 2021. The selected literature is evaluated using the Cochrane System Rating Manual Bias Risk Tool. Statistical analysis and graphics of the inclusion literature are performed using Review Manager 5.3 statistical software.

**Results:**

All the results of this study on the clinical effectiveness and safety of TCM Jinfeng pill in adjuvant treatment of infertility with PCOS will be published in a peer-reviewed academic journal of medicine.

**Ethics and dissemination:**

The type of study is systematic evaluation, the whole process of research does not involve human trials, the data used in the institute are obtained through published literature, so ethical review is not suitable for this study.

**OSF registration number:**

10.17605/OSF.IO/JEP2D. (https://osf.io/jep2d).

**Conclusion:**

Our research will provide evidence-based medical evidence on whether the TCM Jinfeng pill is effective and safe in the treatment of infertility with PCOS.

## 1 Introduction

Polycystic ovarian syndrome (PCOS) is a common endocrine disease in gynecology, which causes infertility in women of childbearing age. Nowadays, the treatment of infertility caused by PCOS has become a hot topic in medical research.^[1,2]^ The clinical characteristics of infertility with PCOS are mainly characterized by occasional ovulation or persistent non-ovulation, and excessive androgen and insulin resistance. Patients may have anovulatory infertility, oligomenorrhea, even amenorrhea, weight gain, thick hair, and other recent clinical manifestations. Most patients are treated for infertility or irregular menstruation. The incidence of infertility caused by this disease is 4% to 10%.^[3]^ At present, the study of infertility with PCOS ethology has not been conclusive, and the ethology of infertility with PCOS has made some discoveries in the fields of genetic correlation, environmental correlation and local molecular biological changes of ovaries.

For the treatment of infertile with PCOS, Western medicine mainly takes drugs (including these types of drugs: ovulation-promoting drugs, drugs to reduce androgen levels, drugs to improve insulin levels, etc.) and surgical treatment, and even the use of assisted reproductive technology. But it has the shortcomings of large side effects, high incidence of adverse reactions and expensive, and drug treatment after discontinuation of the disease is easy to recur.^[4,5]^ In the treatment of infertility with PCOS, TCM regulates the whole body according to TCM theory and treats it based on syndrome differentiation, so as to regulate menstruation and help pregnancy and improve endocrine.^[6]^ Among them, traditional Chinese medicine (TCM) Jinfeng pill has achieved very good clinical results in the treatment of infertility with PCOS.^[7]^ Although there are related literatures in the treatment of infertility with PCOS with TCM Jinfeng pill, the quality of related studies is uneven and there is a lack of strong evidence-based medical evidence. Therefore, this study uses the method of meta-analysis to analyze the clinical effectiveness and safety of Jinfeng pill in the adjuvant treatment of infertility with PCOS, hoping to provide help for the clinical treatment of infertility with PCOS.

## 2 Methods

### 2.1 Protocol registration

The registration information of this protocol in open science framework (OSF) includes: registration number is 10.17605/OSF.IO/JEP2D, the registered website of this protocol is https://osf.io/jep2d.

### 2.2 Inclusion and exclusion criteria for study selection

#### 2.2.1 Types of research

In this study comprehensively collected randomized controlled studies related to the clinical effectiveness and safety of TCM Jinfeng pill in the adjuvant treatment of infertility with PCOS. The retrieval language of database is set to Chinese and English.

#### 2.2.2 Type of participant

The patients diagnosed by clinicians who conformed to infertility with PCOS are selected as the subjects of this trial. Comparing the patient's age, course of illness, and infertility, the difference is not statistically significant and comparable.

##### 2.2.2.1 Inclusion criteria

1. Meet the diagnostic criteria for infertility with PCOS.
2. The patients and their families are aware of this study and have signed an informed consent form.
3. No hormone drugs are used in the 3 months before treatment.
4. The test results of immune and infection indicators are normal.

##### 2.2.2.2 Exclusion criteria

1. Those who with organic disease in the reproductive system.
2. Those who with contraindications to study drugs.
3. Those who are allergic to the study drug.
4. Conference papers with incomplete reports on non-RCT research, reviews, animal experiments, and data.

#### 2.2.3 Type of interventions

The study group is treated with TCM Jinfeng pill. The treatment in the control group can be treated with other traditional Chinese or Western medicine alone, or by combining Jinfeng pill with other TCM or Western medicine. (Both groups of patients take the necessary basic treatment according to the patient's own circumstances, and there are no restrictions on the specification, dosage, medication frequency, and treatment duration of the drug.)

### 2.3 Outcome indicator

#### 2.3.1 Primary outcomes

Overall efficiency and ovarian hemodynamic indexes and endocrine indexes.

#### 2.3.2 Secondary outcomes

Clinical pregnancy rate, ovarian volume, endometrial thickness, the number of follicles on the cut surface, the occurrence of adverse reactions.

### 2.4 Information sources and search strategy

Using the computer to retrieve SinoMed, CNKI, VIP, WANFANG Database, as well as Public, The Cochrane library, Medline (Ovid SP), Embase and other foreign language databases, while manually retrieving the relevant magazine supplements, special issues, professional materials, network information, and so on. Objective to comprehensively collect the randomized controlled trials related to the clinical effectiveness and safety of Jinfeng pill in the adjuvant treatment of infertility with PCOS. The retrieval time is from the beginning of each database to June 2021. The results of search strategy using Embase for literature collection in Table 1.

**Table 1:** Results of search strategy using Embase for literature collection.

Number	Strategy
#1	(Drugs, Chinese Herbal)/exp OR (Chinese Drugs, Plant):ab,ti OR (Chinese Herbal Drugs):ab,ti OR (Herbal Drugs, Chinese):ab,ti OR (Plant Extracts, Chinese):ab,ti OR (Chinese Plant Extracts):ab,ti OR (Extracts, Chinese Plant):ab,ti OR (Chinese Patent Drugs):ab,ti
#2	(Traditional Chinese Medicine)/exp OR (Chung I Hsueh):ab,ti OR (Hsueh, Chung I):ab,ti OR (Traditional Medicine, Chinese):ab,ti OR (Zhong Yi Xue):ab,ti OR (Chinese Traditional Medicine):ab,ti OR (Chinese Medicine, Traditional):ab,ti OR (Traditional Tongue Diagnosis):ab,ti OR (Tongue Diagnoses, Traditional):ab,ti OR (Tongue Diagnosis, Traditional):ab,ti OR (Traditional Tongue Diagnoses):ab,ti OR (Traditional Tongue Assessment):ab,ti OR (Tongue Assessment, Traditional):ab,ti OR (Traditional Tongue Assessments):ab,ti
#3	(Adjuvant Therapy)/exp OR (Drug Therapy, Adjuvant):ab,ti OR (Adjuvant Chemotherapy):ab,ti OR (Adjuvant Drug Therapy):ab,ti OR (Adjuvant Treatment):ab,ti OR (Assistant Therapy):ab,ti
#4	(Jinfeng Pill)/exp
#5	(Polycystic Ovary Syndrome)/exp OR (Ovary Syndrome, Polycystic):ab,ti OR (Syndrome, Polycystic Ovary):ab,ti OR (Stein-Leventhal Syndrome):ab,ti OR (Stein Leventhal Syndrome):ab,ti OR (Syndrome, Stein-Leventhal):ab,ti OR (Sclerocystic Ovarian Degeneration):ab,ti OR (Ovarian Degeneration, Sclerocystic):ab,ti OR (Sclerocystic Ovary Syndrome):ab,ti OR (Polycystic Ovarian Syndrome):ab,ti OR (Ovarian Syndrome, Polycystic):ab,ti OR (Polycystic Ovary Syndrome 1):ab,ti OR (Sclerocystic Ovaries):ab,ti OR (Ovary, Sclerocystic):ab,ti OR (Sclerocystic Ovary):ab,ti
#6	(Infertility)/exp OR (Female Infertility):ab,ti OR (Sterility, Postpartum):ab,ti OR (Postpartum Sterility):ab,ti OR (Subfertility, Female):ab,ti OR (Female Subfertility):ab,ti OR (Sub-Fertility, Female):ab,ti OR (Female Sub-Fertility):ab,ti OR (Sub Fertility, Female):ab,ti OR (Sterility, Female):ab,ti OR (Female Sterility):ab,ti
#7	(#1 OR #2 OR #3) AND #4
#8	#5 AND #6
#9	#7 AND #8

### 2.5 Data collection and analysis

Independently operating by the 2 researchers, according to the inclusion and exclusion criteria established in advance, the literatures retrieve for the first time are screened. After the preliminary screening, the full text is obtained. The data that needs to be extracted include:

1. Basic information of the included literature: author, title, year of publication, etc.
2. Basic information of the research in the literature: sample size of the study, baseline conditions, diagnostic criteria, intervention measures, course of treatment, follow-up time, adverse reactions of the experimental group and the control group.
3. Outcome indicators of the study: total clinical effective number.
4. Information related to the bias evaluation of literature quality.

**Figure 1:**
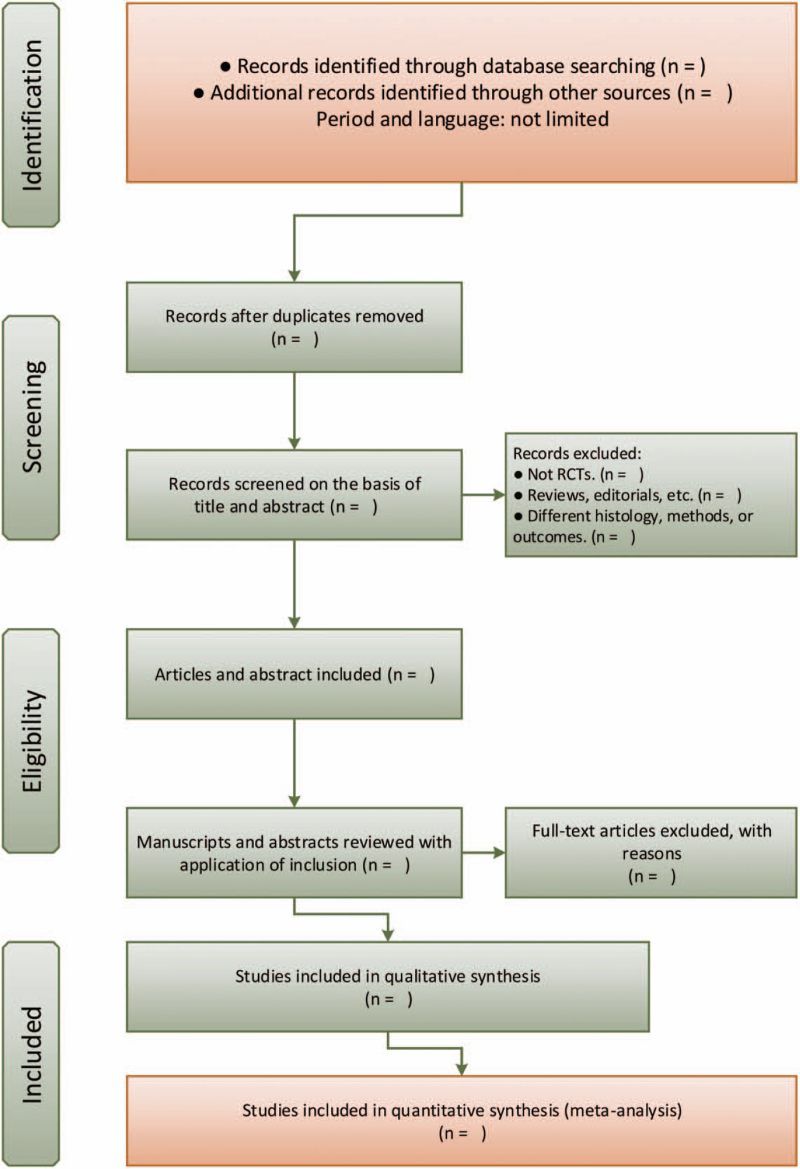
Flow diagram of selection process.

If the data extraction results are inconsistent, the third researcher will intervene in the extraction, and then discussing together to determine the final extracted data. The flow diagram of selection process is shown in Figure 1.

### 2.6 Quality appraisal

This study uses the Cochrane Bias Risk Assessment Tool to evaluate the quality of the included literature. The main contents of the evaluation are 6 aspects, a total of 7 items. According to the judgment basis, the evaluation criteria of each item are divided into “low-risk bias, high-risk bias, unidentified bias.”^[8]^

### 2.7 Data synthesis

The statistical software used in this study meta-analysis is a special system evaluation software review Manager (Review Manager 5.3 statistical software) provided by the Cochrane Collaboration Network, which is recognized by scholars in various countries. The relative risk and its 95% confidence interval (CI) are used for the counting data; the weighted difference mean and its 95% CI are used for metering data. The heterogeneity of each set of data is tested, and if *P* > .05 and *I*^2^ < 50%, the studies are considered to be consistent, using fixed-effect model to analysis. If the consistency between the studies is relatively poor, the random-effects model is used to analyze and the reasons for heterogeneity are analyzed. If statistical analysis is not possible between a certain research indicator, that a descriptive analysis is used. In the case of sensitivity analysis, the data of the research that is significant should be eliminated, the statistical analysis should be analyzed again. Comparing with the results before the exclusion, and if the results obtained before and after are consistent (both different or no differences), the meta-analysis results are more stable, and vice versa, the results are unstable.

### 2.8 Sensitivity analysis

The stability of the results is judged and the sources of heterogeneity are explored. If the included literature is heterogeneous, sensitivity analysis should be performed. By eliminating each case study one by one, the stability of the synthetic effect is compared to determine whether a study is a source of heterogeneity.^[9]^ If it is determined that a study is the source of heterogeneity, it is culled and the source of heterogeneity is analyzed.

### 2.9 Subgroup analysis

The interventions in this study (types of drugs, dosage, frequency and duration of treatment), the age of the patients, and other factors may cause potential publication bias. Therefore, the subgroup analysis will also be carried out from these aspects.

### 2.10 Publication bias

It is often used in funnel plots to evaluate whether the results of meta-analysis are biased. When the amount of included literature is greater than or equal to 10 articles, and it often using inverted funnel analysis. When the funnel plot is asymmetric, it indicates that there is publication bias; when the funnel plot is symmetrical, it means that there is no bias or the degree of bias is small and within an acceptable range.^[10]^

## 3 Discussion

Infertility with polycystic ovary syndrome has polymorphic clinical manifestations. It is an endocrine disease with multiple causes. Its main clinical characteristics are continuous non-ovulation and androgen excess. The women of childbearing age are a high incidence of the population, which is very easy to cause infertility, because of its normal ovulation function of patients caused adverse effects. At present, drug treatment is commonly used for western medicine, including: ovulation drugs, drugs to lower androgen levels, drugs to improve insulin levels and so on. It is a unique advantage that Chinese medicine to treat infertility with PCOS, and few adverse reactions, good long-term effects, which has been widely concerned by scholars. Among them, TCM Jinfeng pill has achieved good results in the treatment of infertility with PCOS. The drug can promote the recovery of ovarian function, promote ovulation and increase the thickness of the endometrium, so as to achieve the goal of increasing the pregnancy rate. The clinical effectiveness of Jinfeng pill to improve infertility with PCOS may be related to the composition of Jinfeng pill. Jinfeng pill is the use of pure Chinese herbs, refined by scientific methods, by thizoma curculiginis, motherwort, ginseng, deer antlers, donkey-hide gelatin, barrenwort, cinnamomum cassia, fructus ligustri lucidi, polygonum multiflorum 9 kinds of Chinese medicine composition. Therefore, Jinfeng pill has the functions of improving hormone disorder, improving immunity, anti-aging, anti-oxidant, hypoglycemic, and lipid-lowering. However, the current deficiency is the lack of strong evidence-based medical evidence for the effectiveness and safety of Jinfeng pill in the adjuvant treatment of infertility with PCOS. Therefore, this study uses meta-analysis method to analyze the clinical effectiveness and safety of Jinfeng pill in the treatment of infertility with PCOS, hoping to provide help for the clinical treatment of infertility with PCOS.

## Author contributions

**Conceptualization:** Lian Hua, Ping Zhu, Jing-Zhi Guan.

**Data curation:** Ping Zhu, Jing-Zhi Guan, Qing-Chun Hai, Jing Jin, Lei Shi.

**Formal analysis:** Ping Zhu, Jing-Zhi Guan.

Funding acquisition: Lian Hua.

**Methodology:** Ping Zhu, Jing-Zhi Guan.

**Resources:** Ping Zhu, Qing-Chun Hai, Jing Jin.

**Software:** Jing-Zhi Guan, Qing-Chun Hai, Lei Shi.

**Writing – original draft:** Ping Zhu, Jing-Zhi Guan, Qing-Chun Hai, Jing Jin, Lei Shi.

**Writing – review & editing:** Lian Hua, Ping Zhu.
